# Neuropsychological profiles and behavioural correlates in individuals exposed to repetitive head impacts: findings from the NEwTON study

**DOI:** 10.1186/s13195-026-02122-4

**Published:** 2026-07-29

**Authors:** Suzie Kamps, Esmé H. J. Habraken, Suzan van Amerongen, Dirk N. van Paassen, Sterre C. M. de Boer, Emma M. Coomans, Sietske A. M. Sikkes, Philip Scheltens, Rik Ossenkoppele, Everard G. B. Vijverberg

**Affiliations:** 1 Alzheimer Center Amsterdam, Neurology, Vrije Universiteit Amsterdam, Amsterdam UMC location VUmc, Amsterdam, the Netherlands; 2 Amsterdam Neuroscience, Neurodegeneration, Amsterdam, the Netherlands; 3 Department of Clinical, Neuro- and Developmental Psychology, VU University, Amsterdam, the Netherlands; 4 Dementia Fund, EQT Life Sciences, Amsterdam, the Netherlands

**Keywords:** Repetitive head impacts, Chronic traumatic encephalopathy, Neuropsychological functioning, Behavioural disinhibition, Traumatic encephalopathy syndrome

## Abstract

**Background:**

Repetitive head impacts (RHI) are associated with an increased risk of neurodegenerative disease, most notably chronic traumatic encephalopathy (CTE). This study characterized neuropsychological profiles of RHI-exposed individuals compared to unexposed controls. To investigate whether cognitive dysfunction is linked to behavioural symptoms in RHI, we examined associations between executive functioning, social cognition and behavioural disinhibition.

**Methods:**

We included RHI-exposed individuals (*N* = 72, 90.4% male, mean age 53.84 ± 12.45) and unexposed, cognitively healthy controls (*N* = 40, 82.5% male, mean age 54.55 ± 12.12) from the “Neurodegeneration: Traumatic brain injury as the Origin of Neuropathology” (NEwTON) study from Amsterdam UMC. Neurocognitive functioning was assessed through a comprehensive test battery covering episodic memory, attention and processing speed, executive functioning, visuospatial abilities, language and semantic memory, and social cognition. Behavioural disinhibition was measured in the RHI group using informant-based questionnaires (the positive items of the Frontal Behavioural Inventory (FBI) and the Neuropsychiatric Inventory (NPI)). Group differences in cognitive domains were assessed with analysis of covariance (ANCOVA) corrected for age, sex and education. The RHI-exposed group was evaluated for Traumatic Encephalopathy Syndrome (TES) criteria (No TES, Suggestive of CTE, Possible CTE, Probable CTE), TES subgroups were exploratively compared on cognitive performance. Associations between behavioural disinhibition and executive functioning or social cognition were assessed with regression analyses.

**Results:**

RHI-exposed individuals performed worse on episodic memory (η^2^ = 0.12, *p*_*FDR*_ ≤ .001), language and semantic memory (η^2^ = 0.10, *p*_*FDR*_ = .001), executive functioning (η^2^ = 0.04, *p*_*FDR*_ = .046), and social cognition (η^2^ = 0.04, *p*_*FDR*_ = .040). No differences were found for visuospatial abilities (η^2^ = 0.01, *p*_*FDR*_ = .221) and, although borderline significant, attention and processing speed (η^2^ = 0.03, *p*_*FDR*_ = .074). Greater TES severity was associated with poorer performance on all cognitive domains. No significant associations were found between executive function or social cognition and behavioural disinhibition, as measured with the NPI and the FBI positive subscale (all *p* > .05).

**Conclusions:**

RHI-exposed individuals showed poorer cognitive performance across several domains (episodic memory, executive functioning, social cognition and language and semantic memory). We found no association between cognitive function and behavioural disinhibition. Further longitudinal studies employing neuroimaging and biomarker data are needed to disentangle the underlying pathology in populations at risk for CTE.

**Supplementary Information:**

The online version contains supplementary material available at https://doi.org/10.1186/s13195-026-02122-4.

## Background

Repetitive head impacts (RHI) are commonly observed in contact sports such as (American) football, rugby, kickboxing or among military personnel exposed to blast injury. These impacts, although often asymptomatic, have been associated with an increased risk of developing the neurodegenerative disease chronic traumatic encephalopathy (CTE) [12, 15, 21]. CTE is characterized by the accumulation of hyperphosphorylated tau (p-tau) in the depths of cortical sulci [20]. As a definitive diagnosis can only be made post-mortem, clinical criteria have been developed for traumatic encephalopathy syndrome (TES) [12]. Individuals conform to TES criteria if they have had substantial exposure to head impacts (i.e. ≥ 5 years), core clinical features (cognitive impairment and/or neurobehavioral dysregulation, and a progressive course) that are not fully accounted for by other disorders. If they conform, individuals can be further classified on the probability of CTE underlying their symptoms (ranging from no CTE to probable CTE [12]. For cognitive dysfunction in TES, deficits in memory and executive functioning domains are considered central, while other cognitive domains are less consistently described.

While studies frequently report cognitive, behavioural, psychiatric and motor symptoms in individuals at risk for CTE, these are often non-specific to CTE, which complicates differentiation from other neurodegenerative disorders [4, 12, 21]. Supporting this, a recent study found no differences in neuropsychological profiles when comparing autopsy confirmed CTE cases to cases with Alzheimer's Disease (AD) [26]. Prior work investigating neuropsychological profiles in former American Football players suggests a diffusely impaired cognitive profile, which involved memory, executive functioning and language, but not visuospatial functions [1]. Yet, published work often focuses on single-sport cohorts, which could introduce cohort effects and limits generalizability to neuropsychological functioning in individuals exposed to RHI from diverse sources.

Behavioural dysregulation such as aggression, impulsivity, irritability and disinhibition, is observed in over 40% of CTE cases [7, 12, 18]. These symptoms can cause significant caregiver burden and induce diagnostic uncertainty, as these symptoms frequently overlap with psychiatric disorders [19]. The link between neurocognitive dysfunction and behavioral change in RHI-exposed individuals remains unclear.

To contribute to earlier detection of cognitive and/or behavioural impairment possibly related to CTE, the present study had a dual aim. First, we assessed to what extent individuals with a history of RHI differed in neuropsychological performance across multiple cognitive domains compared to unexposed controls, and whether cognitive deficits differed between TES classification groups. A comprehensive assessment of neuropsychological functioning following RHI is necessary for assessing risk for CTE in the clinic. Second, we explored whether deficits in executive functioning and social cognition were associated with behavioural disinhibition. This aim was informed by previous findings in AD populations, where deficits in these domains have been linked to neuropsychiatric symptoms such as apathy, anxiety and disinhibition [11, 25, 28]. Identifying these relationships may help explain neurobehavioural dysregulation in individuals at risk for CTE and improve clinical recognition of CTE-related cognitive-behavioural profiles.

## Methods

### Participants

Participants were recruited from the “Neurodegeneration: Traumatic brain injury as the Origin of Neuropathology” (NEwTON) study, a prospective cohort study in Amsterdam, the Netherlands, designed to assess the brain health of RHI-exposed individuals at risk for CTE [31]. Recruitment occurred through several pathways, specified in the previously published study protocol. Mainly, participants were either recruited via the memory clinic at the Amsterdam Alzheimer Centre in (*N* = 27) or through sources such as sports federations or advertisements (*N* = 45).

Inclusion criteria for the RHI group were: (1) aged 30 or older, (2) a history of exposure to RHI through contact sports or military service involving blast exposure, and (3) a minimum of six years of exposure, in accordance with published work [12]. Further, participants were excluded if they had sustained a concussion or severe TBI within the year preceding testing, to minimize the effects of acute TBI. In the RHI group, participants were symptomatic, as changes in cognition, mood or behaviour were reported by either themselves or their environment. A diagnosis of a neurodegenerative disease was not an exclusion criteria, as this does not exclude presence of (comorbid) CTE pathology.

Healthy controls were recruited through Hersenonderzoek.nl, an online recruitment platform for people interested in participating in brain research [36]. Controls were also required to be at least 30 years of age and were excluded if they had actively participated in contact sports, any history of RHI, any other head trauma, or if they had reported neurological, psychiatric or neurodegenerative conditions. The control group and the RHI-group were age-and-sex matched. Age matching was done by matter matching to age tertiles of the RHI group.

The study was approved by the Medical Ethical Review Committee (METC; P2021-011) of Amsterdam UMC and was conducted in accordance with the principles of the Declaration of Helsinki and Dutch legislation on medical research involving human subjects (WMO). Written informed consent was obtained from all participants.

### Procedure

Participants in the RHI group underwent a comprehensive assessment protocol including a neuropsychological assessment, medical and collateral history interviews, neurological examinations, MRI scan, blood sampling, optionally a lumbar puncture for cerebrospinal fluid (CSF) analysis and several questionnaires for sleep quality and cognitive change. Informant-based questionnaires were administered to close relatives. Following the assessment, a multidisciplinary team evaluated whether the participant with RHI met the TES criteria [12]. The classifications of the TES criteria are: no CTE, suggestive of CTE, possible CTE or probable CTE. The level of functional dependence and supportive features, such as motor symptoms and psychiatric symptoms, were also noted [12]. In 2 participants (2.8%) TES classification could not be applied, because the clinical presentation was inconclusive and no study partner could be reached for verification of clinical or behavioural symptoms. Amyloid beta positivity was based on CSF analysis for amyloid beta 42 (Aβ42) and hyperphosphorylated tau at threonine 181 (p-tau181). Predefined cut-off values for Aβ42 of < 813 pg/mL [29], until June 2018 or < 1092 pg/mL [34] from June 2018 onward, were used as well as a p-tau181/Aβ42 ratio of > 0.020 [33]. This information was available for 35 individuals (46% of RHI), as not all participants agreed to a lumbar puncture. Seven individuals in the RHI group, recruited from the memory clinic, had also undergone an amyloid PET scan. An experienced nuclear physician visually assessed the scan for amyloid positivity.

Control participants completed a limited assessment including the same neuropsychological test battery, a brain MRI scan, and blood sampling. No informant-based questionnaires were administered in the control group and for this group no information on amyloid-status was available.

#### Neuropsychology

Neuropsychological domains assessed included episodic memory, executive functioning, social cognition, attention and processing speed, language and semantic memory and visuospatial ability. An extensive description of the neuropsychological domains and the corresponding individual tests are provided in the Supplementary Material. In short, episodic memory consisted of the Visual Association Test (VAT), the Auditory Verbal Learning Test (AVLT) and the Rey Complex Figure Test (RCFT) delayed recall condition. The Executive Functioning (EF) domain consisted of the Digit Span Backwards test, Letter-Fluency test, Trail-Making-Test part B (TMT-B), and the colour-word (inhibition) condition of the Stroop test. Language and semantic memory were evaluated using the Animal Fluency test. Attention and processing speed was made up of the Digit Span Forward test (WAIS-IV), the Nummer Letter Substitution Test (NLST), the Trail Making Test part A (TMT-A) and the reading and colour naming conditions of the Stroop Colour-Word test. Visuospatial abilities were assessed using the copy condition of the RCFT and two subtests from the Visual Object and Space Perception Battery (VOSP): Fragmented Letters and Number Location. Social cognition was assessed with the Ekman 60 faces test for emotion recognition. Missing data were observed across the cognitive domains, with 2 cases missing for the domain executive functioning (2.7% in RHI, 0% in control), 3 cases for attention and processing speed (4.1% in RHI, 0% in control), 4 cases for episodic memory (5.5% in RHI, 0% in control), 4 cases for visuospatial abilities (5.5% in RHI, 0% in control), 2 cases for language (2.7% in RHI, 0% in control) and 7 cases for social cognition (4.1% in RHI, 10% in control). Results are based on complete cases, as missing values were not imputed.

#### Behavioural disinhibition

Behavioural disinhibition was measured using two informant-based questionnaires: the Frontal Behavioural inventory (FBI) and the Neuropsychiatric Inventory (NPI). With the FBI, a study partner close to the participant was asked to rate the presence and severity of frontal behavioural symptoms in the participant. For each item, responses ranged from 0 (never) to 3 (frequently). Only the positive items, which are related to disinhibited behaviour, were included in the current study, based on prior work identifying their relevance to frontotemporal dementia [13]. These items included: alien hand syndrome, perseveration, irritability, inappropriate humour, poor judgement, socially inappropriate behaviour, impulsivity, restlessness, aggression, hyperorality, hypersexuality, utilization behaviour and incontinence. The maximum total score was 39 points.

The NPI assessed additional neuropsychiatric symptoms including symptoms of NBD through structured interviews with a close informant [6, 8]. When a symptom was endorsed, follow-up questions assessed frequency (scored 1–4) and severity (scored 1–3). The total score was calculated by multiplying frequency by severity for each item and summing across relevant domains. The maximum total score was 144 points. The NPI score was missing for four individuals (6%) and the FBI score for seven individuals (10%) of the RHI group.

### Statistical analysis

To enable comparison across tests within each cognitive domain, raw scores were converted into z-scores. Normative values were derived from the control group and used to compute standardized scores for each participant. For each domain, a mean z-score was calculated by averaging the z-scores of the corresponding tests. For all tests, higher scores indicated better performance, except for the Trail Making Test (TMT) and the Stroop Colour-Word Task, these z-scores were therefore inversed prior to computing the composites. The control group of the NEwTON study performed the same test battery, allowing for direct comparison on complete cognitive domains which are presented as main analyses, but is relatively small in sample size. The Amsterdam Dementia cohort (ADC) includes 440 healthy controls screened at the Amsterdam Alzheimer Center without cognitive impairment or amyloid pathology [32]. The available neuropsychological tests largely overlap with those in the NEwTON study, with the exception of the NLST, RCFT, and the Ekman 60 faces test. As the ADC sample size is larger and therefore more suitable as a reference group for z-transformation for these tests, we used the means and standard deviations of these healthy controls to create domain composites with the available tests in sensitivity analyses. These are presented in the Supplementary Material.

We assessed group differences (RHI vs controls) on the cognitive domain composite scores (episodic memory, EF, attention and processing speed, language, visuospatial function, and social cognition) using Analysis of Covariance (ANCOVA) controlled for age, sex and education. We adjusted the significance for the false discovery rate (FDR). For each dependent variable we tested assumptions for ANCOVA prior to analysis. We checked the linearity between the continuous covariate (age) and the dependent variables with visual inspection of scatterplots. Homogeneity of regression slopes was assessed by including an interaction term of group * age on the dependent variables. Normality of residuals was tested using the Shapiro–Wilk test. Homoscedasticity of residuals was assessed with Levene’s test on the model’s residuals. Significant outliers were identified using standardized residuals (>|3|) and Cook’s distance (Cook’s D > 4/*N*). Violations of assumptions were addressed by transforming variables and/or running sensitivity analyses without outliers where appropriate. Transformation was done according to the boxcox function in R (package “MASS”) indicating the appropriate transformation per variable that makes up the domain score. In case this did not resolve the violation of assumptions, we also performed the non-parametric Kruskal–Wallis test and reported these in addition to the ANCOVA results. In addition, although amyloid positivity was not an exclusion criteria for the study, a sensitivity analysis was performed to account for potential influence of amyloid positivity on the results. ANCOVA analyses were repeated after excluding participants from the RHI group that were amyloid positive (*N* = 7). We also repeated the ANCOVA tests exploratively to investigate group differences in the cognitive domains between TES subgroups (no TES, suggestive of CTE, possible CTE, probable CTE).

Associations between the behavioural measures (FBI and NPI) and cognitive measures (executive function and social cognition z-scores) were investigated with linear regressions.. Regressions were controlled for age and sex.

P-values below 0.05 indicated statistical significance. For the ANCOVA, partial eta squared (*η*^2^) was examined to assess effect size. Effect sizes were small (*η*^2^ = 0.01–0.06), medium (η^*2*^ = 0.06–0.14) or large (*η*^2^ > 0.14). In the regression analyses, the standardized Beta coefficient (*β*) was used to interpret effect size. Values between *β* = 0.30—0.49 indicated a moderate effect, and *β* ≥ 0.50 reflected a large effect [23].

## Results

### Descriptive statistics

The sample consisted of 112 participants, consisting of a group with RHI exposure (*N* = 72) and a control group without RHI exposure (*N* = 40). Analysis of demographic characteristics revealed no significant group differences in ages, education level or sex (Table 1). The main source of RHI exposure in the RHI group was fighting sports (45%), followed by soccer (23%), rugby (16%), military (10%), or classified as ‘other’ (6%). Of the individuals exposed to RHI and classified as TES, there were 13 that had cognitive impairment only, 13 had NBD only, and 10 individuals had both. Seven participants in the RHI group were diagnosed with AD. Of these, six had CSF measurements positive for amyloid pathology, and one individual had a positive amyloid PET scan without CSF data. In addition, six individuals had a negative amyloid PET scan. Two of these also had CSF measurements (both negative), while four did not have CSF data, resulting in four additional amyloid-negative individuals based on PET. In total, this resulted in 32 amyloid-negative individuals in the RHI group.

**Table 1:** Participant Demographics

	**RHI** **(*N* = 72)**	**Controls** **(*N* = 40)**	**Statistics**
Age in years (mean ± SD)	53.84 ± 12.45	54.55 ± 12.12	*F* (111) =.19, *p* =.666
Level of education (Verhage, 1964)^a^(%)			*X*^*2*^ (4) = 7.81, *p* =.099
Verhage ≤ 5	43.7%	20.0%	
Verhage 6	39.7%	52.5%	
Verhage 7	16.4%	27.5%	
Sex (%)			*X*^*2*^ (1) = 1.49, *p* =.222
Male	90.4%	82.5%	
Female	9.6%	17.5%	
MMSE (mean ± SD)	27.73 ± 2.58	29.43 ±.68	*F* (111) = 32.41, *p* = < *.001*
TES Classification (%)			
*Missing*	2.8%		
No TES	46.6%		
TES- Cognitive impairment	32.9%		
TES—Neurobehavioral dysregulation	32.9%		
TES – level of functioning			
No dementia			
Mild functional impairment	64.4%		
Mild dementia	20.5%		
TES—Motor symptoms	15.1%		
TES—Psychiatric symptoms	6.8%		
Degree of exposure	31.5%		
Substantial (> 5 year)	17.8%		
Extensive (> 11 year)	82.2%		
Provisional level of certainty of CTE pathology (%)			
Suggestive of CTE	17.8%		
Possible CTE	16.4%		
Probable CTE	16.4%		
NPI total score (mean ± SD)	9.72 ± 13.91		
FBI total score (mean ± SD)	11.54 ± 11.21		
Positive items total (mean ± SD)	4.29 ± 4.29		
Negative items total (mean ± SD)	7.25 ± 7.62		

*TES* Traumatic Encephalopathy Syndrome, *CTE* chronic traumatic encephalopathy, *MMSE* Mini-Mental State Examination, *FBI* Frontal Behavioral Inventory, *NPI* Neuropsychiatric Inventory

^*a*^Verhage ≤ 5: pre-vocational education (LTS), lower general secondary education (MAVO), intermediate vocational education (MBO); Verhage 6: higher general secondary education (HAVO), pre-university education (VWO); Verhage 7: higher professional education (HBO)

### Assumptions

There was no deviation from linearity observed. The interaction term group*age was significant when predicting episodic memory (*p* < 0.001) and language (*p* = 0.045), therefore homogeneity of regression slopes was not met. Inspection of the interaction revealed the group difference to increase with increasing age, which suggest a potential exacerbation of cognitive deficits in these domains related to age in the RHI group. The Shapiro–Wilk test was significant for episodic memory (*p* ≤ 0.001), executive function (*p* ≤ 0.001), attention and processing speed (*p* ≤ 0.001), and visuospatial function (*p* ≤ 0.001), for these variables normality of residuals was violated. Levene’s test was significant for episodic memory (*p* = 0.036), indicating homogeneity of variances was not met. For each of episodic memory, attention and processing speed, visuospatial function and executive function, significant outliers were identified. The violated assumptions were tested again with transformed variables and without significant outliers, this did not yield better outcomes. Therefore, the original data was used in the analyses. We performed additional non-parametric Kruskal–Wallis tests to assess group differences in the cognitive domains, as a way to control the results for the unmet assumptions of ANCOVA. As these models cannot be controlled for covariates, they are reported as supportive analyses.

### Group differences in neurocognitive functioning

**Fig. 1:**
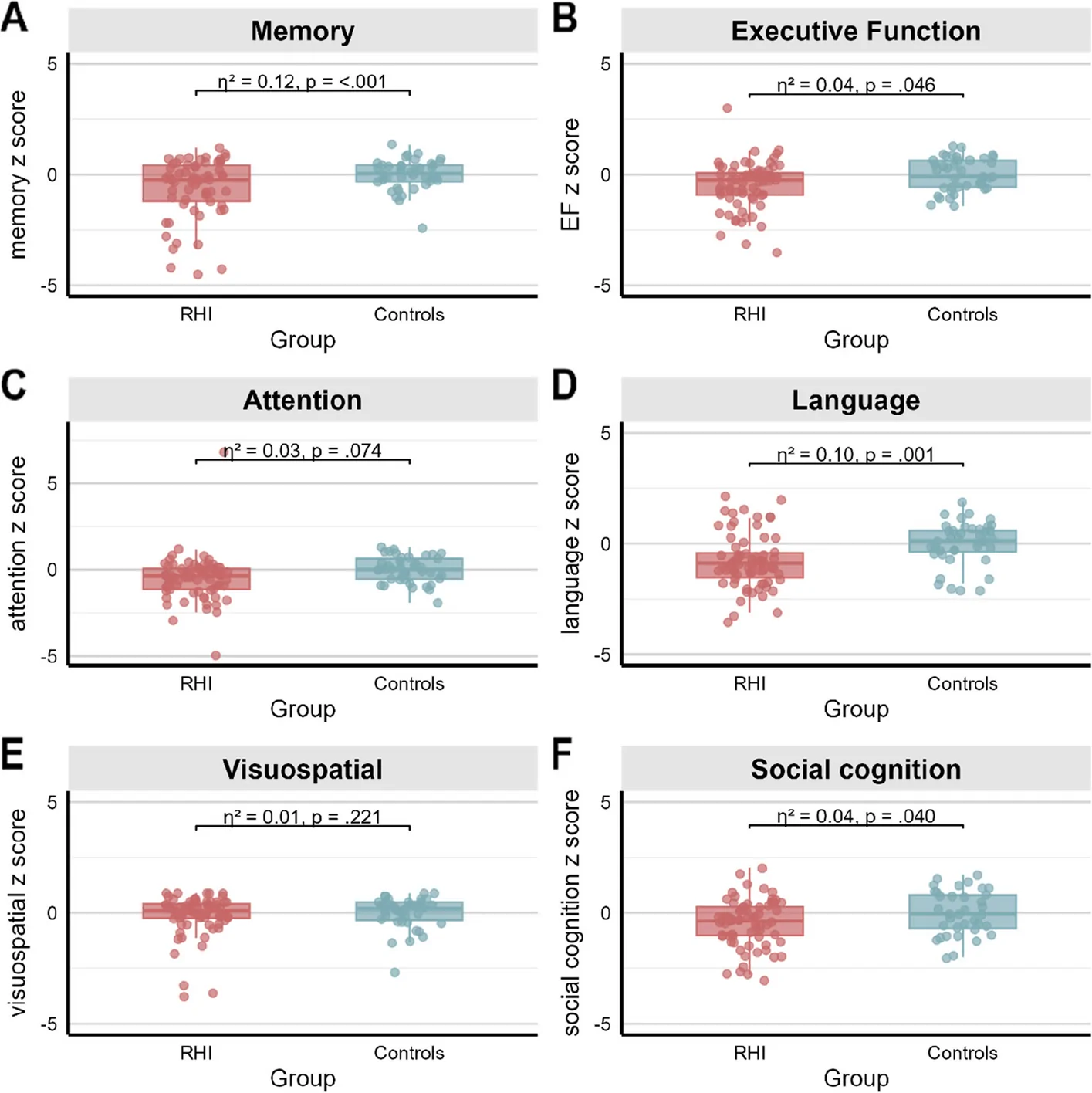
Boxplots depicting group differences (RHI vs. controls) in the different neurocognitive domains (**A**: Episodic Memory; **B**: Executive Functioning; **C**: Attention and Processing Speed; **D**: Language and Semantic Memory; **E**: Visuospatial Functioning; **F**: Social Cognition). Y-axes represent z scores, all participants are referenced to the control group. The statistics presented in the figure are results from the ANCOVA analyses corrected for age, sex and education. RHI = Repetitive Head Impacts, EF = Executive Functioning

Group differences in neurocognitive function are represented in Table 2 and Fig. 1. The RHI-exposed individuals performed significantly worse on the domain composites of episodic memory (*η*^2^ = 0.12, *F* = 14.26, *p*_*FDR*_ ≤ 0.001), executive functioning (*η*^2^ = 0.04, *F* = 4.10, *p*_*FDR*_ = 0.046), language and semantic memory (*η*^2^ = 0.10, *F* = 11.38, *p*_*FDR*_ = 0.001), and social cognition (*η*^2^ = 0.04, *F* = 4.32, *p*_*FDR*_ = 0.040). Effect sizes were in the small to medium range, with medium effects observed for episodic memory and language, and small effects for executive functioning and social cognition. There was no difference for attention and processing speed, although borderline significant (*η*^2^ = 0.03, *F* = 3.27, *p*_*FDR*_ = 0.074), neither for visuospatial function (*η*^2^ = 0.01, *F* = 1.52, *p*_*FDR*_ = 0.221). Non-parametric Kruskal–Wallis tests showed similar outcomes, except for attention and processing speed which was now also significantly lower for the RHI-exposed group (*χ2* = 8.13, *p* = 0.004), see Supplementary Table 1.

**Table 2:** ANCOVA results for between-group differences (RHI-exposed group vs controls)

	***F***	***η2***	***p***_***FDR***_
Episodic memory			
Corrected (age, sex, education)	14.26	.12	<.001***
Corrected (age, sex, education, A^+^ excluded)	11.49	.11	.002**
Executive functioning			
Corrected (age, sex, education)	4.10	.04	.046*
Corrected (age, sex, education, A^+^ excluded)	2.11	.02	.149
Social cognition			
Corrected (age, sex, education)	4.32	.04	.040*
Corrected (age, sex, education, A^+^ excluded)	2.93	.03	.090
Attention and processing speed			
Corrected (age, sex, education)	3.27	.03	.074
Corrected (age, sex, education, A^+^ excluded)	1.56	.02	.214
Language and semantic memory			
Corrected (age, sex, education)	11.38	.10	.001***
Corrected (age, sex, education, A^+^ excluded)	8.39	.08	.005**
Visuospatial functioning			
Corrected (age, sex, education)	1.52	.01	.221
Corrected (age, sex, education, A^+^ excluded)	0.13	.00	.718

*RHI* Repetitive Head Impacts, *A*^*+*^ amyloid positive (7 individuals within RHI group)

^*^p <.0.05, ** *p* <. 01, *** p <.001

**Fig. 2:**
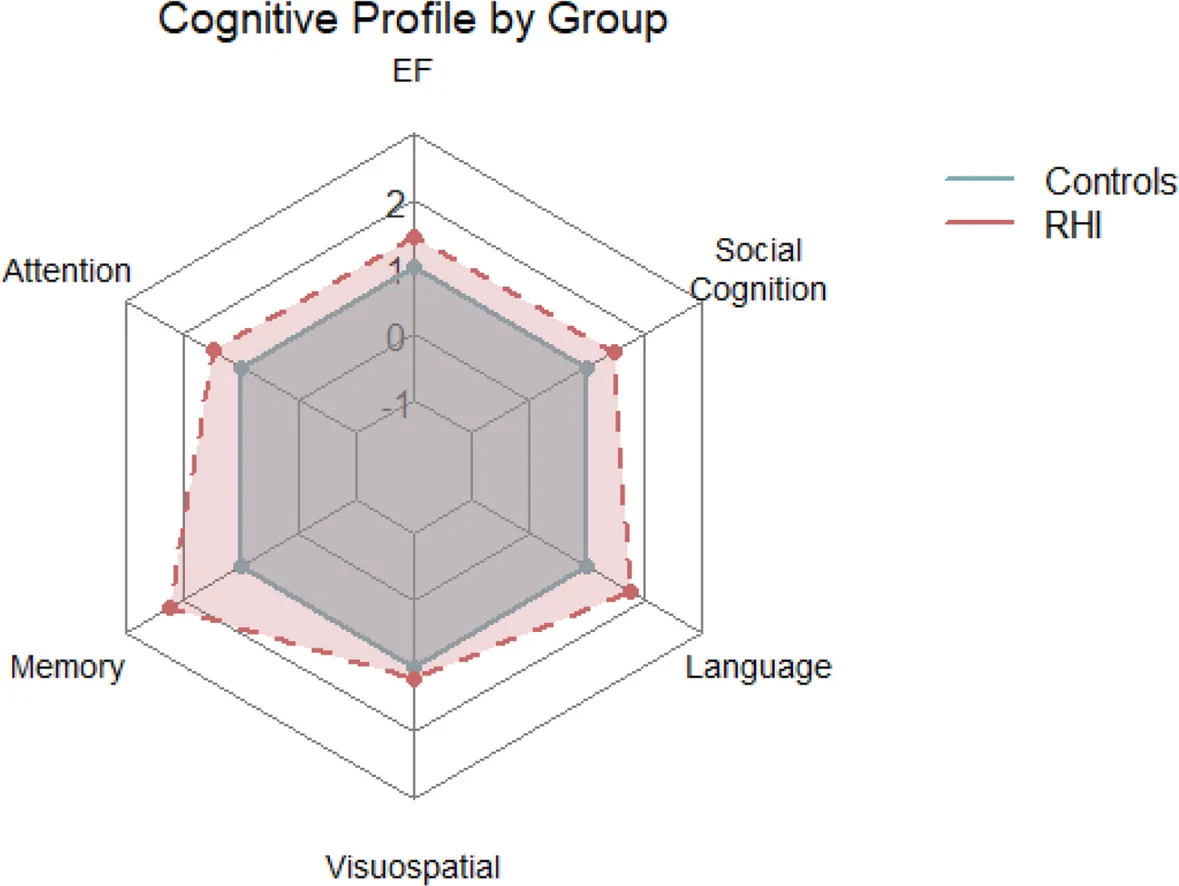
Radar plot showing the neurocognitive profiles of the different groups (RHI in red vs controls in blue). The values −2; + 2 represent absolute mean z scores, which are referenced to the control group. The coloured surfaces represent the proportion (%) of observations within that deviation of the mean. Z scores are inverted for visualization purposes, the more outward means the greater negative deviation from zero. The plot shows the groups to differ on all neurocognitive domains except for visuospatial function. RHI = Repetitive Head Impacts, EF = Executive Function

In Fig. 2 the neuropsychological profiles based on domain composites are represented in a radar plot, in Supplementary Fig. 1 a radar plot shows all individual tests per group. As shown in the radar plots, RHI-exposed individuals perform worse across neurocognitive domains except for visuospatial functioning, with the most apparent difference in memory.

### Sensitivity analyses

A sensitivity analysis was conducted after excluding those in the RHI group that were amyloid positive (*N* = 7). There were no differences in age, sex, functional impairment or TES metrics between those with (*N* = 35) and without (*N* = 37) a lumbar puncture (all *p* > 0.05), which was assessed to control for potential selection bias. Significant group differences persisted for the domains episodic memory (*η*^2^ = 0.11, *F* = 11.49, *p*_*FDR*_ = 0.002) and language (*η*^2^ = 0.08, *F* = 8.39, *p*_*FDR*_ = 0.005). Group differences in executive function (*η*^*2*^ = 0.02, *F* = 2.11, *p*_*FDR*_ = 0.149) and social cognition (*η*^2^ = 0.03, *F* = 2.93, *p*_*FDR*_ = 0.090) were no longer significant. Similar results were seen in the additional non-parametric tests, see Supplementary Table 1, and when standardizing test scores for the healthy ADC controls, Supplementary Material.

### Differences between TES subgroups

**Fig. 3:**
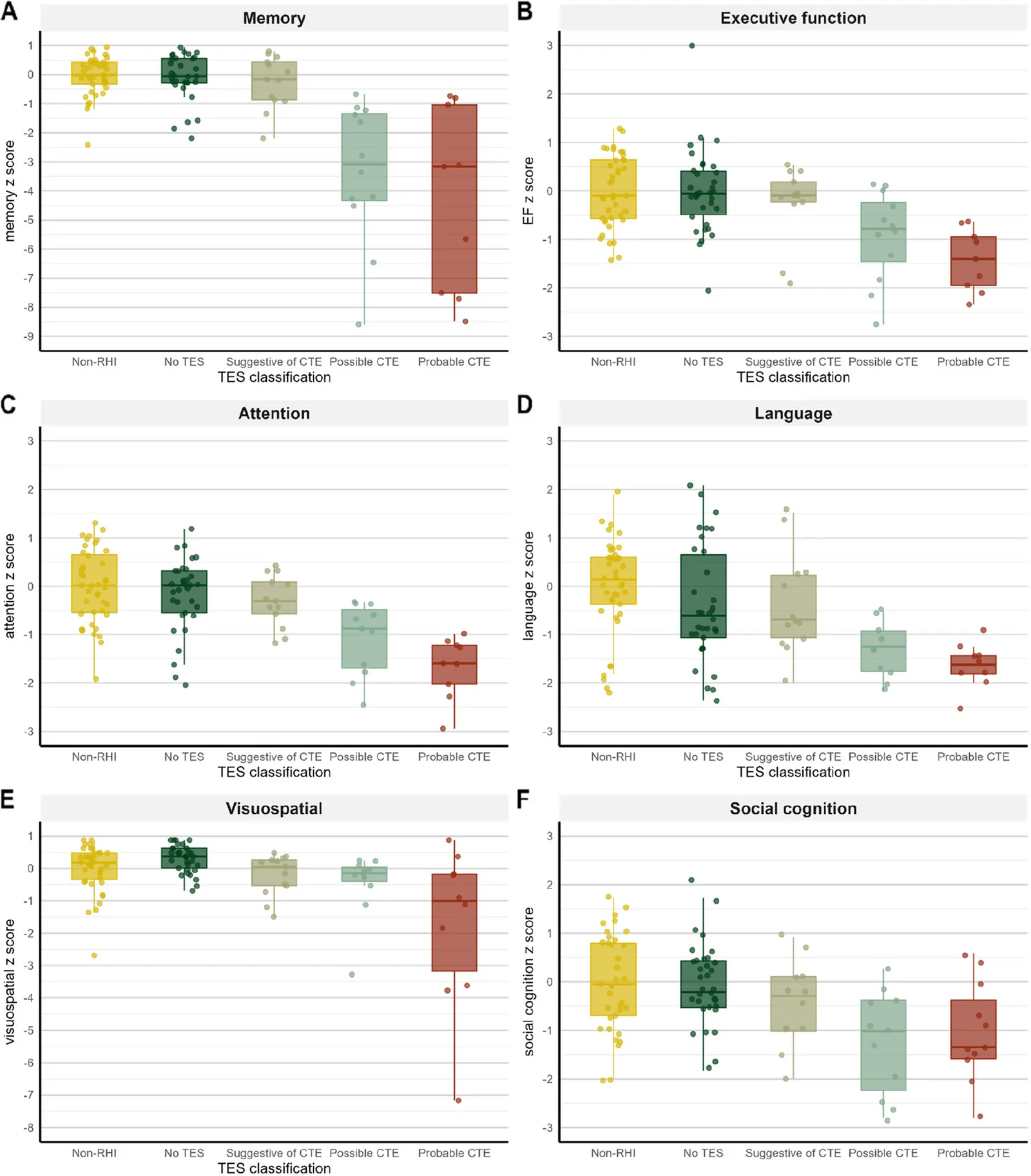
Boxplots depicting group differences (RHI vs. controls) in the different neurocognitive domains, split on the different TES subgroups (**A**: Episodic Memory; **B**: Executive Functioning; **C**: Attention and Processing Speed; **D**: Language and Semantic Memory; **E**: Visuospatial Functioning; **F**: Social Cognition). Non-RHI individuals were not included in the analysis, but are shown in the plot for informative purposes. Y-axes represent z scores, all participants are referenced to the control group. All domains differed significantly between groups. RHI = Repetitive Head Impacts, EF = Executive Functioning; TES = Traumatic Encephalopathy Syndrome

Exploratively, ANCOVA’s were conducted within the RHI-group, investigating differences in cognitive domain composites between TES-subgroups: no CTE, suggestive CTE, possible CTE and probable CTE (Table 3, Fig. 3). The subgroups performed significantly different on all cognitive domains, decreasing cognitive performance was seen for increasing CTE likelihood.

**Table 3:** ANCOVA analyses comparing the TES subgroups (age, sex, education included as covariates)

	***F***	***η*2**	***p***
Episodic memory^b,c,d,f^	11.91	.37	<.001***
Executive functioning^b,c,e,f^	9.15	.30	<.001***
Social cognition^b,c^	4.16	.17	.009**
Attention and processing speed^b,c,f^	8.11	.29	<.001***
Language and semantic memory^c,f^	3.62	.15	<.018*
Visuospatial functioning^c,e,f^	5.88	.23	.001**

TES Traumatic Encephalopathy Syndrome, TES classified subgroups in the RHI-group: no TES, suggestive of CTE, possible CTE, probable CTE. Contrasts a,b,c,d,e,f indicate which groups differ significantly from each other. a = No TES – Suggestive of CTE, b = No TES – Possible CTE, c = No TES – Probable CTE, d = Suggestive of CTE – Possible CTE, e = Possible CTE – probable CTE, f = Suggestive of CTE – Probable CTE

^*^p <.0.05, ** *p* <. 01, *** p <.001

### Behavioural correlates

**Fig. 4:**
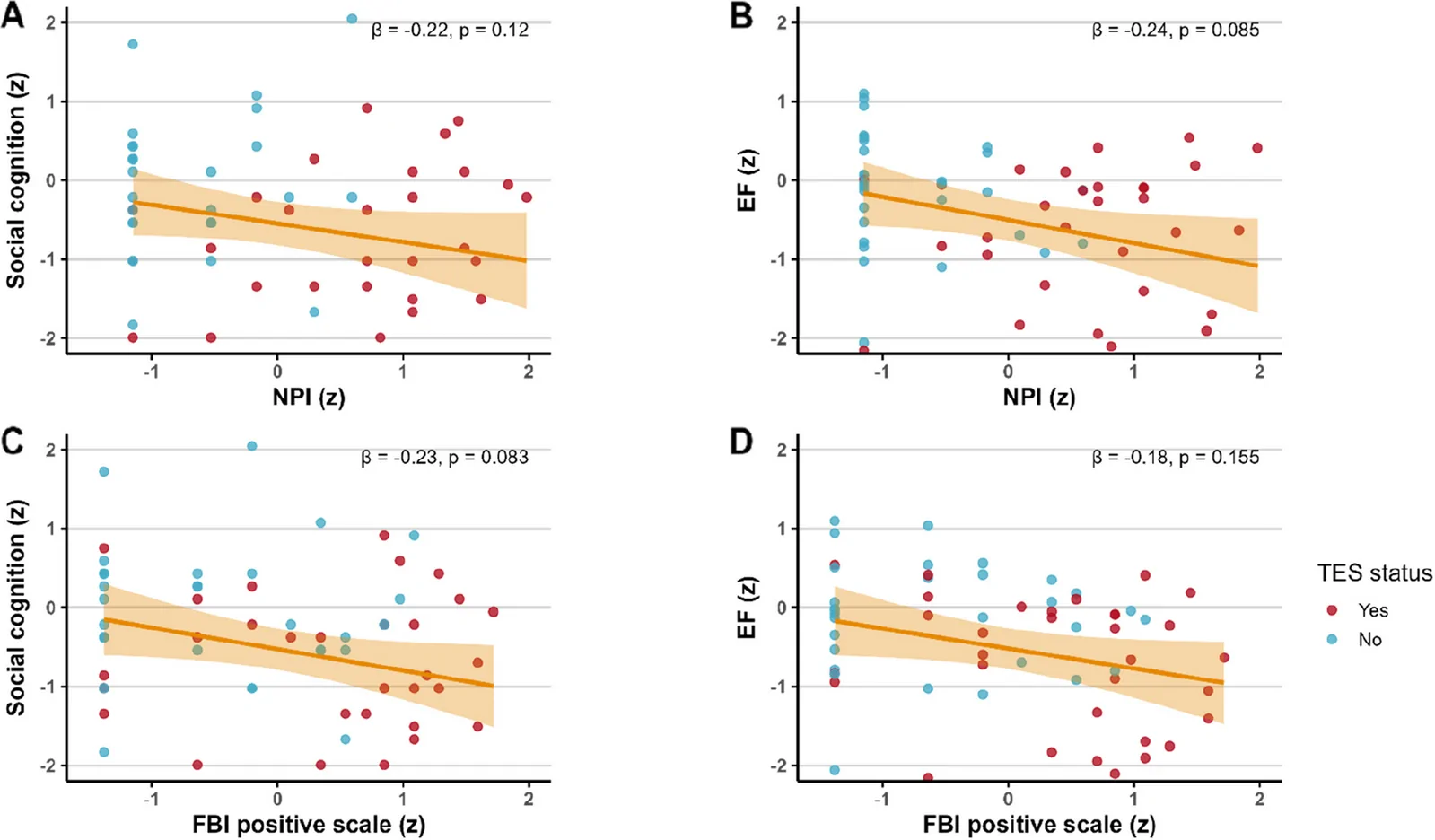
Plots showing associations between neuropsychiatric symptoms and cognitive outcomes (**A**, **B**) and frontal behavioral symptoms and cognitive outcomes (**C**, **D**) through linear models within the RHI group. The NPI results were available for 65 individuals, and the FBI results for 68 individuals. The data points are coloured for adhering to TES criteria (yes vs no) for visualisation purposes, those that are classified as TES generally have more behavioural symptoms. RHI = Repetitive Head Impacts. TES = Traumatic Encephalopathy Syndrome. NPI = Neuropsychiatric Inventory. FBI = Frontal Behavior Inventory

The regression analyses showed no significant associations between scores of the NPI and social cognition (β = −0.22, *p* = 0.120, Fig. 4A) or executive functioning (β = −0.24, *p* = 0.085, Fig. 4B). Similarly, no significant associations were found between scores of the FBI positive subscale and social cognition (β = −0.23, *p* = 0.083, Fig. 4C) or executive functioning (β = −0.18, *p* = 0.155, Fig. 4D.

## Discussion

The aim of the present study was to characterize neurocognitive functioning in individuals at increased risk of developing CTE. Specifically, we examined whether individuals with a history of RHI performed differently on neuropsychological measures compared to a control group without such exposure. The RHI-exposed group showed poorer performance across the domains of episodic memory, executive functioning, social cognition, and language and semantic memory. For attention and processing speed, those with RHI exposure had lower scores than controls, the difference was however non-significant. Sensitivity analyses excluding individuals in the RHI-group that were amyloid-positive revealed that the group differences remained significant for episodic memory and language and semantic memory. Exploratory analyses revealed significant differences between TES subgroups on all cognitive domains, with moderate to large effect sizes. Individuals classified as probable CTE performed worse across domains, while those without TES showed the best performance. Overall, we found a diffusely impacted profile of neurocognitive functioning, with the most pronounced differences between those with RHI and controls in episodic memory, executive functioning and language and semantic memory.

The moderate group differences found in neurocognitive functioning are consistent with prior findings indicating that deficits in episodic memory and executive functioning are hallmark features of TES [12, 22]. Furthermore, the present results show that individuals with RHI exposure also performed worse on measures of language and semantic memory, and (marginally) attention and processing speed, these domains have been less frequently studied in the context of CTE. For former American Football players, earlier research has found the memory domain to be disproportionally impaired compared to other domains, therefore proposing the neuropsychological profile to mimic that of amnestic MCI [1, 24]. In the current cohort of individuals exposed to mixed causes of RHI, most individuals did not have formal cognitive deficits as established by normative neuropsychological assessment, yet there is a diffuse pattern of deficits in several cognitive domains. This is consistent with a recently published clinical review, describing retired contact sports athletes to present with cognitive complaints in memory, concentration, and word-finding, with relative sparsity of neurological symptoms or impaired daily life functioning [10]. In contrast, performance in visuospatial functioning did not differ significantly between groups, suggesting that specific cognitive processes may be less vulnerable to the effects of RHI. A possible explanation is that visuospatial tasks primarily involve occipital and parietal brain regions, while RHI is more commonly associated with neuron loss in the frontotemporal areas [3, 16]. Overall, the results highlight that RHI can affect cognitive domains beyond memory and executive functioning, even after excluding individuals with biomarker evidence of amyloid pathology.

Although the initial analyses showed a significant group difference in social cognition and executive function, these effects were no longer significant when excluding individuals with amyloid positivity, indicating that these may have substantially contributed to the group effect. A prior study found plasma p-tau181 and p-tau217, markers of AD pathology, able to identify AD pathology in patients with TES, but limited utility in TES patients without AD [5]. Generally, AD biomarkers are thought to be useful in at risk CTE individuals to rule in or rule out AD pathology, but not for identifying CTE tau [4]. Notably, RHI are associated with both AD [9, 30] and with CTE [21]. Further, amyloid pathology is commonly observed, as amyloid beta plaques have been found in 50% of cases with autopsy-confirmed CTE [27], and CTE can be a common contributing pathology when AD is most likely the primary disease driving the symptoms [14]. For the 32 RHI-exposed individuals without biomarker data, the prevalence of amyloid positivity is unknown, which is acknowledged as a limitation. In our sample, 33 out of 40 individuals with available CSF and/or PET data were negative for amyloid pathology, suggesting that the observed cognitive profile cannot be solely attributed to AD pathology. Therefore, while it is unknown what the exact underlying pathologies in our study sample are, the results of the sensitivity analysis indicate amyloid can exacerbate neurocognitive deficits, rather than fully explaining them. Future studies with longitudinal measures of biomarkers for neurodegenerative processes and clinical outcomes could help disentangle which disease primarily contributes to which symptoms.

Contrary to expectations, no significant associations were found between executive functioning or social cognition and behavioural disinhibition. This contrasts with studies in AD populations, where reduced cognitive functioning has been linked to neuropsychiatric symptoms such as apathy, irritability, and disinhibition [11, 25]. A possible explanation is the relatively low prevalence of behavioural disinhibition in the RHI group in the current sample. Scores on questionnaires for neuropsychiatric and behavioural symptoms were generally low and skewed to the right, suggesting a potential floor effect that could obscure associations. This interpretation aligns with findings from a study in which associations between social cognition and apathy were absent in patients with mild cognitive impairment or neurodegenerative conditions other than AD [28]. Behavioural symptoms may be more sensitively captured with measures assessing behavioural functioning across daily life. It is plausible however that individuals with more severe neuropsychiatric or cognitive symptoms would also be more difficult to recruit. Additionally, behavioural disinhibition was only measured in the RHI group, precluding comparison with the control group. Altogether, it is possible that behavioural disinhibition emerges in more advanced stages of neurodegeneration, which may explain its absence in this relatively young cohort. Nevertheless, earlier work with American Football players, aged around 50 years old, showed younger age of first exposure to be related to more neurobehavioral and cognitive complaints [2]. These contrasting findings could relate to the difference in head impacts in the current sample compared to American Football. Lastly, the non-significant finding could indicate behavioural disinhibition be more strongly influenced by other factors than executive functioning or social cognition in this cohort, i.e. personality characteristics, lifestyle factors or other cognitive functions. This remains to be further explored.

This study has several limitations. First, the cross-sectional design prevents causal inferences. It remains unclear to which extent the observed cognitive differences are attributable to RHI or to other factors. For example, sensitivity analyses excluding amyloid-positive individuals showed that two of the four domains lost statistical significance. There were no systematic differences in those with and those without a lumbar puncture, nevertheless the influence of amyloid positivity cannot be fully captured due to the unknown rate of amyloid positive individuals in the full sample. Future studies, ideally with longitudinal designs, could help identify which individuals convert from subjective to objective cognitive complaints, and moreover which pathology they develop. Second, participants in the RHI group were symptomatic as they were recruited based on reported changes in cognition or behaviour, whereas controls were selected based on the absence of neurological or psychiatric diagnoses. This recruitment strategy may introduce circularity and selection bias, as individuals in the RHI group were already concerned about their functioning. Consequently, the observed group differences cannot be interpreted as causal effects of RHI exposure per se. However, the primary aim of the study was to characterize neuropsychological profiles within an at-risk group, rather than to establish the presence or absence of cognitive impairment attributable to RHI exposure. Third, it is important to note that nearly half of the RHI group did not meet TES diagnostic criteria. This variability may have diluted detectable patterns of subtle cognitive decline. Fourth, a definitive diagnosis of CTE cannot be made during life, meaning that the cohort represents individuals at risk for CTE without having pathological confirmation of CTE diagnosis. The NEwTON study has a brain donation program, therefore in the future the clinical data can be interpreted in the context of which individuals develop CTE pathology, and which do not. Lastly, not all assumptions for ANCOVA were met, which could not be improved by transforming the data or removing outliers. Although the groups did not differ significantly in age, sex, or education, these factors were still deemed important to include in the models, therefore performing non-parametric tests without possibility of adding covariates was not preferable. However, we did perform non-parametric Kruskal–Wallis tests in addition to the ANCOVA’s. These yielded similar results, which adds robustness to our findings.

Despite these limitations, the current study also has several important strengths. The variability in head impacts of the sample is notable: participants included both amateur and professional contact sport athletes across a variety of sports, whereas previous studies have often focused on a single sport or population [17, 35]. This diversity enhances the generalizability of the findings and provides a broader understanding of the cognitive effects of RHI exposure.

Based on the present findings, several recommendations can be made for future research and clinical practice. Longitudinal studies incorporating neuroimaging techniques and biomarkers are essential to examine the progression of neurocognitive symptoms and their relation to neuropathology over time. Moreover, examining longitudinal trajectories in neuropsychological functioning could help establish the prognostic value of these assessments for individuals at risk of CTE, providing insight into their likelihood of developing a neurodegenerative disease. Although the current study cannot declare the cause of the observed cognitive impairments, clinicians should remain alert to a history of RHI exposure in patients presenting with cognitive complaints, as this may be a contributing factor.

## Conclusions

In summary, individuals with RHI exposure exhibited worse performance on tasks assessing episodic memory, executive functioning, social cognition, and language and semantic memory compared to unexposed controls. In individuals with RHI, no significant associations were found between executive functioning or social cognition and behavioural disinhibition, potentially due to low prevalence of behavioural symptoms in the sample. Future research using longitudinal data, neuroimaging, and biomarkers is needed to further our understanding of neuropsychological functioning and relation to disease progression of RHI-related neurodegeneration. This study contributes to the growing body of literature by delineating the neuropsychological characteristics of individuals at risk for CTE, where we highlight the importance of recognizing subtle deficits in neuropsychological functioning and the complexity of a diffusely impacted neurocognitive profile.

## Supplementary Information

Supplementary Material 1

## Data Availability

The dataset used and analysed in the current study can be made available upon reasonable request.

## Abbreviations

TBI: Traumatic Brain Injury
CTE: Chronic Traumatic Encephalopathy
RHI: Repetitive Head Impacts
FBI: Frontal Behaviour Inventory
NPI: Neuropsychiatric Inventory
TES: Traumatic Encephalopathy Syndrome
AD: Alzheimer’s Disease
EF: Executive Functioning
TMT: Trail Making Test
VOSP: Visual Object and Space Perception Battery
WAIS: Wechsler Adult Intelligence Scale
ALVT: Auditory Verbal Learning Test
NLST: Number Letter Substitution Test
NBD: Neurobehavioural Dysregulation
