# Supplementary material for: Neuropsychological profiles and behavioural correlates in individuals exposed to repetitive head impacts: findings from the NEwTON study
Source: Alzheimers Res Ther. 2026 Jul 29;18:237. doi: 10.1186/s13195-026-02122-4 (PMC13711815; doi:10.1186/s13195-026-02122-4)
Supplement: Supplementary file 1 [file 13195_2026_2122_MOESM1_ESM.docx]

**Neuropsychological profiles and behavioural correlates in individuals exposed to repetitive head impacts: findings from the NEwTON study**

**Supplementary Material**

Contents

1. Methods extended: Neuropsychological tests
2. Results extended
3. Supplementary Table 1
4. Supplementary Figure 1
5. Supplementary Figure 2

**Methods extended: Neuropsychological tests**

***Episodic memory***

Episodic memory was assessed using the Visual Association Test (VAT), the Auditory Verbal Learning Test (AVLT) and the Rey Complex Figure Test (RCFT) delayed recall condition after three minutes. For the VAT, the score reflected the number of correctly recalled image pairs (Meyer & de Jonghe, 2019). The AVLT involved five immediate recall trials, with a total score ranging from 0 to 75 (Saan & Deelman., 1986; Egberink & Lang., 2024). The delayed recall took place after 20 to 25 minutes, where participants were asked to recall the words again, the score ranging from 0 to 15 points. Recognition was assessed by presenting target and distractor words, with a maximum score of 30 points. In the RCFT delayed recall condition, participants were asked to reproduce the previously copied figure from memory after a 3-minute interval (Rey, 1941; Egberink & Lang., 2024). Scoring follows the standard protocol used in the copy condition.

***Executive functioning***

Executive functioning was assessed using the Digit Span Backwards test, Letter-Fluency test, Trail-Making-Test part B (TMT-B), and the colour-word condition of the Stroop test. For the Digit Span Backwards test, the number of correctly recalled sequences was summed, with a maximum score of 21 (Lindeboom & Matto, 1994). In the Letter Fluency task, participants were asked to generate as many words as possible in one minute beginning with the letters “D”, “A” or “T” in separate trials (Van Der Elst et al., 2006). The final score consists of the total number of correct responses. The TMT-B required participants to alternately connect numbers and letters in ascending order, and the score consists of the time in seconds to complete the task (Tombaugh, 2003). For the Stroop test, the score consists of the number of seconds required to correctly name the ink colour of incongruent colour words (Stroop, 1992; Egberink & Lang., 2024).

***Social cognition***

Social cognition, specifically the emotion recognition component of social cognition, was assessed using the Ekman 60 Faces Test (Dutch version) (Kessels & Montagne, 2016). This test required participants to identify facial expressions representing six emotions: happiness, fear, disgust, anger, sadness and surprise. Each emotion was presented 10 times, and the total score ranging from 0 to 60 points represented the number of correct identifications across the items.

***Attention and processing speed***

This domain was assessed using the Digit Span Forward test (WAIS-IV), the Number Letter Substitution Test (NLST), the Trail Making Test part A (TMT-A) and the reading and colour naming conditions of the Stroop Colour-Word test. The Digit Span Forward score was calculated as the total number of correctly repeated sequences, with a maximum score of 21 (Lindeboom & Matto, 1994; Egberink & Lang., 2024; Wechsler., 2008). In the NLST, participants matched letters to corresponding digits as quickly as possible, and the score reflected the number of correct substitutions completed within 90 seconds (Van Der Elst et al., 2006). TMT-A required participants to connect numbers in ascending order, the score reflected the time taken in seconds (Tombaugh, 2003). The Stroop test, condition 1 measured the reading speed and condition 2 measured colour naming, in both cases the score was the total time in seconds to complete the task (Stroop, 1992).

***Language and semantic memory***

Language and semantic memory were evaluated using the Category Fluency task, condition animals. Participants were asked to name as many animals as possible within one minute, the score was the total number of correct animal names generated (Van Der Elst et al., 2006; Egberink & Lang., 2024; Zhao et al., 2013).

***Visuospatial functioning***

Visuospatial abilities were assessed using the copy condition of the RCFT and two subtests from the Visual Object and Space Perception Battery (VOSP): Fragmented Letters and Number Location. The RCFT copy condition required participants to reproduce a complex figure and the performance score was based on the accuracy and placement of the elements of the figure with a score ranging from 0 to 36 points (Rey, 1941). The VOSP subtests were scored based on the number of correct responses for each condition (Warrington & James, 1995; Dağli et al., 2023).

**Results extended**

The ANCOVA analyses were also repeated for the slightly less extensive domain composites with test scores standardized for the ADC healthy controls (*N*=440). These yielded similar results, with the domain attention and processing speed additionally being significant between groups (*η*²=0.06, *F*=7.00, *p_FDR_*=.009).

| **Supplementary Table 1.** Non parametric Kruskal-Wallis tests comparing RHI and controls | | | |
| --- | --- | --- | --- |
| **Domain** | | **χ2** | ***p* value (FDR corrected)** |
| Episodic memory | | 7.70 | **.005**** |
|  | A^+^ excluded | 5.10 | **.024*** |
| Executive functioning | | 5.36 | **.021*** |
|  | A^+^ excluded | 3.58 | .059 |
| Social cognition | | 3.87 | **.049*** |
|  | A^+^ excluded | 2.80 | .094 |
| Attention and processing speed | | 8.13 | **.004**** |
|  | A^+^ excluded | 6.20 | .**013*** |
| Language and semantic memory | | 13.62 | **<.001***** |
|  | A^+^ excluded | 10.80 | **.001**** |
| Visuospatial functioning | | 0.42 | .521 |
|  | A^+^ excluded | 0.08 | .780 |
| RHI = Repetitive Head Impacts. A^+^ = amyloid positive. FDR = false discovery rate  **p* < .0.05, ** *p* <. 01, *** p < .001 | | | |

**Supplementary Figure 1.** Radar plot showing the performance on individual tests of the different groups (RHI in red vs controls in blue). The values -2;+2 represent absolute mean z scores, which are referenced to the control group. The coloured surfaces represent the proportion (%) of observations within that deviation of the mean. Z scores are inverted for visualization purposes, the more outerward means the greater negative deviation from zero. RHI = Repetitive Head Impacts, EF = Executive Function; VOSP=Visual Object and Space Perception Battery; NLST=Number Letter Substitution Test; TMT=Trail Making Test; VAT=Visual Association Test; ALVT=Auditory Verbal Learning Test
